# Impact of sarcopenia on daily functioning: a cross-sectional study among older inpatients

**DOI:** 10.1007/s40520-022-02175-z

**Published:** 2022-07-06

**Authors:** Dominic Bertschi, Caroline M. Kiss, Nadine Beerli, Oliver Mauthner, Reto W. Kressig

**Affiliations:** 1 University Department of Geriatric Medicine FELIX PLATTER, Basel, Switzerland; 2 Department of Geriatrics, Inselspital, Bern University Hospital, University of Bern, Bern, Switzerland; 3 Department of Public Health, Institute of Nursing Science, University of Basel, Basel, Switzerland

**Keywords:** Sarcopenia, Functional status, Cognition, Geriatrics, Older adults

## Abstract

**Background and aim:**

Geriatric patients with sarcopenia are at increased risk for functional decline with loss of independence in daily living. This cross-sectional study aims to investigate the impact of sarcopenia on different domains of functional status in hospitalized geriatric patients.

**Methods:**

Sarcopenia was assessed at hospital admission using the recommendations of the European Working Group on Sarcopenia in Older People 2 (EWGSOP2). Body impedance analysis (BIA) was performed to determine muscle mass, and a pneumatic hand dynamometer was used to assess muscle strength. The functional independence measure (FIM) score, an 18-item tool exploring an individual's physical, cognitive and social functions, was used to measure functional status.

**Results:**

In 305 included inpatients with a median age of 84.0 years (65.6% female), prevalence of sarcopenia was 22.6%. Overall, sarcopenic patients had significant lower FIM scores compared to non-sarcopenic patients (*p* = 0.006). An association with sarcopenia was found for the FIM items bed/chair/wheelchair transfer (*p* = 0.047), transfer to toilet (*p* = 0.048), locomotion (*p* = 0.001), climbing stairs (*p* = 0.012), comprehension (*p* = 0.029), and social interaction (*p* = 0.028).

**Conclusion:**

In hospitalized geriatric patients, sarcopenia was found to be associated with both cognitive and mobility domains, but not with self-care domains of the FIM score. Therefore, when addressing sarcopenia in inpatients, tailored and multi-dimensional training interventions mainly should focus on motor-cognitive abilities.

**Supplementary Information:**

The online version contains supplementary material available at 10.1007/s40520-022-02175-z.

## Introduction

Sarcopenia, a prevalent clinical syndrome in older patients defined as loss of muscle mass, strength and function [1], has been recognized as an independent disease with an International Classification of Disease-10 code [2]. Although a universal definition is still lacking, sarcopenia is associated with negative clinical outcomes such as poor mobility and increased mortality [3–5].

Loss of muscle mass and strength also poses an increased risk of functional impairment which can lead to loss of independence in performing activities of daily living [6]. Findings from different studies show that sarcopenia is associated with worse recovery of physical functioning after stroke and lowers the rate of discharge in a home environment setting, indicating that sarcopenia  decisive factor with regard to whether or not a patient will be dependent on the help of another person in everyday life [7–10]. The most recent guideline from the European Working Group on Sarcopenia in Older People 2 (EWGSOP2) underlines the relevance of functional status in the modern concept of sarcopenia by defining low muscle strength as a key characteristic of this clinical syndrome [11].

To assess a person’s ability to perform activities of daily living, studies frequently use the functional independence measure (FIM) score. This 18-item, seven level, ordinal scaled tool evaluates an individual’s physical, cognitive and social functions [12]. Although different subdomains of the FIM score such as motor and cognitive domains have been evaluated in sarcopenic patients, so far no study has investigated the association of sarcopenia with the different 18 items in older inpatients. A separate analysis of all items, however, would allow an assessment of the different domains of functional status in relation to sarcopenia.

The current study aims to assess the impact of sarcopenia on daily functioning in a population of hospitalized geriatric patients. We hypothesize that different domains of daily functioning are associated with sarcopenia.

## Methods

### Participants and setting

This study included a consecutively recruited sample of 305 patients older than 65 years admitted to an acute geriatrics unit and a geriatric rehabilitation unit of a University department for medicine of aging between September 10 and October 30, 2019. The standardized rehabilitation program included a total of 300 min/week of physiotherapy, occupational and nutritional therapy for patients admitted to the acute care setting, and a total of 450 min/week for patients admitted to the rehabilitation unit. Before the study was conducted, all participants provided written informed consent. The consent form was signed by an authorized proxy person when the patient was unable to give consent due to severe cognitive impairment. Exclusion criteria were inability to follow study procedures, acute sepsis, parameters interfering with body impedance analysis (e.g., severe dehydration or volume overload), and an estimated life expectancy of < 3 months.

At hospital admission, all study participants underwent a geriatric assessment in addition to a baseline geriatric examination, which included the German version of the mini mental state exam (MMSE) [13], the timed up and go test (TUG) [14], the nutritional risk screening (NRS 2002) [15], and the functional independence measure (FIM) score [12]. Moreover, all participants were assessed for sarcopenia within the first 6 days of admission using recommendations of the European Working Group on Sarcopenia in Older People 2 (EWGSOP2) [11]. To determine muscle mass, body impedance analysis (BIA 101, Akern, Florence, Italy) was performed. A pneumatic hand dynamometer (Martin Vigorimeter^®^, Gebrüder Martin GmbH, Tuttlingen, Germany) was used to assess muscle strength. Cut-off points for low handgrip strength were < 50 kPa for men and < 34 kPa for women > 75 years old, and < 64 kPa for men and < 42 kPa for women ≤ 75 years old [16]. Sarcopenia was diagnosed when both handgrip strength and muscle quantity were low [11].

The study was approved by the Ethics Committee of Northwest and Central Switzerland (registration ID 2019-01461) and registered at ClinicalTrials.gov (NCT04124575).

### Functional independence measure

To assess functional status of all participants at hospital admission, trained nurses and physiotherapists used the functional independence measure (FIM) score. The 18 items included in this tool can be further summarized into six domains: Self-care (6 items), sphincter control (2 items), transfers (3 items), locomotion (2 items), communication (2 items) and social cognition (3 items). The score of each item ranges from 1 to 7 points, with higher scores reflecting a better functionality. The total FIM score therefore ranges from 18 to 126 points and is not specific to any diagnosis.

### Statistical analysis

Categorical variables were reported as frequencies (n) and percentages (%), and continuous variables as medians and 25–75th interquartile ranges (IQR). Sample size calculation of this cross-sectional study was previously reported [17]. Functional status (total FIM score) of sarcopenic and non-sarcopenic patients was compared using Mann–Whitney *U* test. In a secondary analysis, sarcopenic and non-sarcopenic patients were assessed according to four FIM quartiles: 99–126 points (1. quartile), 72–98 points (2. quartile), 45–71 points (3. quartile), and 18–44 points (4. quartile). These quartiles were selected a priori by the research team based on clinical experience since there are no existing FIM score cut-offs for definition of low functional status in the literature. Pearson-Chi-square test and Fisher’s exact test were used, where appropriate. The association of each FIM item with sarcopenia was assessed in a multivariate logistic regression model including the parameters age and sex. Statistical analysis was performed using SPSS Statistics, Version 22 (IBM SPSS Statistics, Chicago, IL). *p* values < 0.05 were considered to be statistically significant.

## Results

Clinical characteristics of the study population are described in Table 1 and elsewhere [17]. Median age of the 305 included patients was 84.0 years (IQR 79.0–89.0 years), and 65.6% were female. Median body mass index (BMI) was 25.6 kg/m^2^ (IQR 22.3–28.9 kg/m^2^), and 54.1% of the study population were at risk of malnutrition. Main reasons for hospital admission were orthopedic (38.0%), neurological (28.2%), infectious (15.4%) and cardiovascular (9.2%) diseases. Other diseases reflected 9.2% of hospital admissions. Prevalence of sarcopenia was 22.6% (CI 17.9–27.3%), and median total FIM score was 76 points (62–90 points).

**Table 1:** Clinical characteristics of the study population (*n* = 305)

General characteristics	
Age, years, median (IQR)	84 (79–89)
Women, *n* (%)	200 (65.6)
BMI, kg/m^2^, median (IQR)	25.6 (22.3–28.9)
Calf circumference, cm, median (IQR)	32.5 (30.0–35.0)
Mid-arm circumference, cm, median (IQR)	26.5 (23.5–29.0)
Confirmed sarcopenia, *n* (%)	69 (22.6)
Number of drugs, median (IQR)	7 (5–10)
Number of comorbidities, median (IQR)	5 (4–6)
Length of hospital stay, days, median (IQR)	16 (12–21)
Functional status (FIM)	
Total FIM score, points, median (IQR)	76 (62–90)
Self-care	
Feeding, points, median (IQR)	6 (5–6)
Grooming, points, median (IQR)	5 (4–6)
Bathing/showering, points, median (IQR)	3 (1–4)
Dressing upper body, points, median (IQR)	4 (3–6)
Dressing lower body, points, median (IQR)	3 (1–5)
Toileting, points, median (IQR)	4 (3–6)
Sphincter control	
Bladder management, points, median (IQR)	5 (4–6)
Bowel management, points, median (IQR)	5 (5–6)
Transfer	
Bed, chair, wheelchair, points, median (IQR)	5 (3–6)
Toilet, points, median (IQR)	5 (3–6)
Tub or shower, points, median (IQR)	2 (1–4)
Locomotion	
Walking/wheelchair, points, median (IQR)	5 (2–6)
Stairs, points, median (IQR)	1 (1–1)
Communication	
Comprehension audio/visual, points, median (IQR)	5 (5–6)
Expression verbal/non-verbal, points, median (IQR)	6 (5–6)
Social cognition	
Social interaction, points, median (IQR)	5 (4–6)
Problem solving, points, median (IQR)	4 (3–5)
Memory, points, median (IQR)	5 (4–6)

BMI, body mass index; FIM, functional independence measure; IQR, interquartile range

### Functional status of sarcopenic and non-sarcopenic patients

**Fig. 1:**
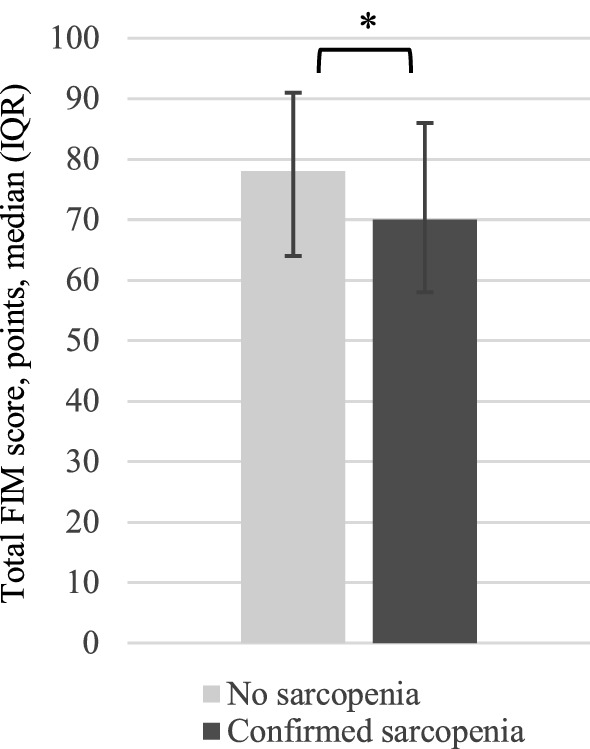
Functional status of sarcopenic and non-sarcopenic hospitalized geriatric patients. *Significant difference (*p* < 0.05) between the groups “no sarcopenia” and “confirmed sarcopenia”. FIM, functional independence measure

Functional status of sarcopenic and non-sarcopenic patients is showed in Fig. 1. Overall, sarcopenic patients had significant lower total FIM scores compared to non-sarcopenic patients [70 points (IQR 58–86 points) and 78 points (IQR 64–91 points), respectively, *p* = 0.006]. Supplemental Fig. 1 depicts the distribution of sarcopenic and non-sarcopenic patients classified into four quartiles in relation to total FIM score. Patients in the lowest FIM quartile were more likely to be sarcopenic than non-sarcopenic (13.0% and 5.9%, respectively; *p* = 0.049). Consistent with this finding, non-sarcopenic patients were found more often in the highest quartile compared with sarcopenic patients (10.6% and 1.4%, respectively; *p* = 0.013). No significant differences were found in the second and third quartile between sarcopenic and non-sarcopenic patients.

### Association of FIM score characteristics with sarcopenia

The association of the different FIM items with sarcopenia, adjusted for age and sex, are presented in Table 2. Overall, sarcopenia was associated with lower total FIM scores (OR 1.02; CI 1.00–1.03). An association was also found for the FIM items bed/chair/wheelchair transfer (OR 1.19; CI 1.00–1.42), transfer to toilet (OR 1.18; CI 1.00–1.40), locomotion (OR 1.30; CI 1.12–1.51), climbing stairs (OR 1.52; CI 1.10–2.12), comprehension (OR 1.28; CI 1.02–1.59), and social interaction (OR 1.27; CI 1.03–1.57). In contrast, no association with sarcopenia was found for the other FIM items.

**Table 2:** Association of the FIM items with sarcopenia

FIM domain	Characteristic	OR (95% CI)*	*p* value
Self-care	Feeding	1.23 (0.99–1.51)	0.059
	Grooming	1.09 (0.92–1.31)	0.380
	Bathing/showering	1.05 (0.88–1.24)	0.609
	Dressing upper body	1.06 (0.92–1.23)	0.423
	Dressing lower body	1.04 (0.90–1.21)	0.582
	Toileting	1.14 (0.98–1.34)	0.099
Sphincter control	Bladder management	1.12 (0.97–1.30)	0.128
	Bowel management	1.15 (0.95–1.39)	0.140
Transfer	Bed, chair, wheelchair	1.19 (1.00–1.42)	*0.047*
	Toilet	1.18 (1.00–1.40)	*0.048*
	Tub or shower	1.01 (0.86–1.18)	0.939
Locomotion	Walking/wheelchair	1.30 (1.12–1.51)	*0.001*
	Stairs	1.52 (1.10–2.12)	*0.012*
Communication	Comprehension audio/visual	1.28 (1.02–1.59)	*0.029*
	Expression verbal/non-verbal	1.23 (0.99–1.52)	0.063
Social cognition	Social interaction	1.27 (1.03–1.57)	*0.028*
	Problem solving	1.14 (0.93–1.40)	0.201
	Memory	1.17 (0.96–1.41)	0.118

FIM, functional independence measure; SD, standard deviation

*p* values < 0.05 were considered to be statistically significant

*Logistic regression, adjusted for age and sex

## Discussion

This study shows that geriatric inpatients diagnosed with sarcopenia had significant lower scores of functional status at hospital admission compared to non-sarcopenic patients. Multiple items of the FIM domains of social cognition, communication, locomotion and transfer were found to be associated with sarcopenia, whereas no association was found for all items of the FIM domains of self-care and sphincter control.

To the best of our knowledge, this is the first study to assess the 18 items of the FIM score and their association with sarcopenia in a large sample of hospitalized geriatric patients. Previous studies have shown that FIM scores were lower in sarcopenic patients admitted to rehabilitation wards compared to non-sarcopenic individuals [7]. Furthermore, it has been reported that a lower FIM score is independently associated with sarcopenic obesity in older patients with stroke [18]. In contrast to our data, none of these studies provides information on all items of the FIM score in older inpatients. In our study, we were able to include a substantial number of older hospitalized patients and to evaluate the association of sarcopenia with the different domains of daily functioning.

Our finding of sarcopenia being associated with low scores of different items of the FIM domains social cognition and communication reflects the current literature [19–22]. One theory that might help to explain the link between sarcopenia and cognitive impairment is based on chronic excessive inflammation and oxidative stress that are related to both conditions. It was found that elevated levels of inflammatory markers such as C-reactive protein (CRP) and Interleukin-6 (IL-6) are associated with loss of skeletal muscle mass [23], strength [24] and an increased risk for dementia [25]. Another plausible theory is a cross-talk between muscle and brain that is mediated by exercise-induced myokine release [26]. Myokines are muscle-derived molecules with positive autocrine, paracrine and endocrine effects on cell proliferation and muscle hypertrophy [27]. They are also known to enhance the function of the central nervous system [28]. Following this theory, it can be hypothesized that limited physical activity in sarcopenic patients might lead to a reduced myokine release, and that lower levels of myokines might contribute to a cognitive decline. However, although this hypothesis is coherent with the findings of our study, one must also take into consideration that the link between muscle and brain seems to be very complex. More research is needed to fully understand the underlying processes involved in this association.

The results of our study indicate that cognitive impairment should always be considered when treating sarcopenia in hospitalized patients. In addition to routinely performed diagnostic measures, multi-dimensional interventional approaches focusing on mobility and cognition should be implemented. Due to the chronicity of both sarcopenia and cognitive impairment, these multi-dimensional interventions should begin right after hospital admission, and continue after hospital discharge.

Further research is needed to evaluate if timely diagnostic measures and an early start of tailored interventions might not only help reducing sarcopenia and poor physical function, but also contribute to stabilize or even improve cognitive function. In a randomized controlled trial including 44 older post-stroke patients with sarcopenia, a combined eight-week intervention of a leucine-enriched amino acid supplementation and low-intensity resistance training significantly increased muscle mass, strength and physical function [29]. However, the intervention and observation time of eight weeks in this study was too short to find any significant change in FIM cognitive score. Therefore, large interventional studies with a sufficient longitudinal design are needed to evaluate the effect of nutrition intervention and physical exercise on cognitive outcomes in sarcopenic patients.

This study has limitations. This single-center study of Caucasian geriatric inpatients in a wealthy country is based on a cross-sectional design in which causal relationships remain unclear. However, we were able to recruit a substantial consecutive sample of geriatric hospitalized patients. Longitudinal multicentre-studies are needed to assess functional outcomes of sarcopenic geriatric inpatients and treatment options. Larger sample sizes will also allow to adjust regression models for more possible confounders than age and sex. Another limitation of this study is the fact that the assessment of the FIM score has been performed by various assessors. However, all assessors were skilled nurses and physiotherapists receiving regular training sessions. Compared to other comprehensive geriatric assessment instruments such as the MMSE, the FIM score is an observation-based instrument that includes only limited information on different domains of functional status. The advantage of using the FIM score is to work with a simple tool that can be integrated in clinical daily life of different patient settings.

## Conclusion

In geriatric hospitalized patients, sarcopenia has been found to be associated with different domains of the FIM score, especially with impaired cognition and mobility. To address sarcopenia in hospitalized geriatric patients, timely diagnosis as well as tailored and multi-dimensional interventions that include nutritional, mobility and cognitive components need to be considered.

## Supplementary Information

Below is the link to the electronic supplementary material.

Supplementary file1 (DOCX 69 KB)

## Data Availability

All data presented in this study are reported in the article.

## Declarations

### Statement of human and animals rights

The study was approved by the Ethics Committee of Northwest and Central Switzerland (registration ID 2019-01461) and registered at ClinicalTrials.gov (NCT04124575).
