# Supplementary material for: Impact of sarcopenia on daily functioning: a cross-sectional study among older inpatients
Source: Aging Clin Exp Res. 2022 Jul 6;34(9):2041–6. doi: 10.1007/s40520-022-02175-z (PMC9464162; doi:10.1007/s40520-022-02175-z)
Supplement: Supplementary file 1 — Supplementary file1 (DOCX 69 KB) [file 40520_2022_2175_MOESM1_ESM.docx]

**Supplemental Figure 1.** Distribution of sarcopenic and non-sarcopenic hospitalized geriatric patients according to four quartiles of total FIM sore


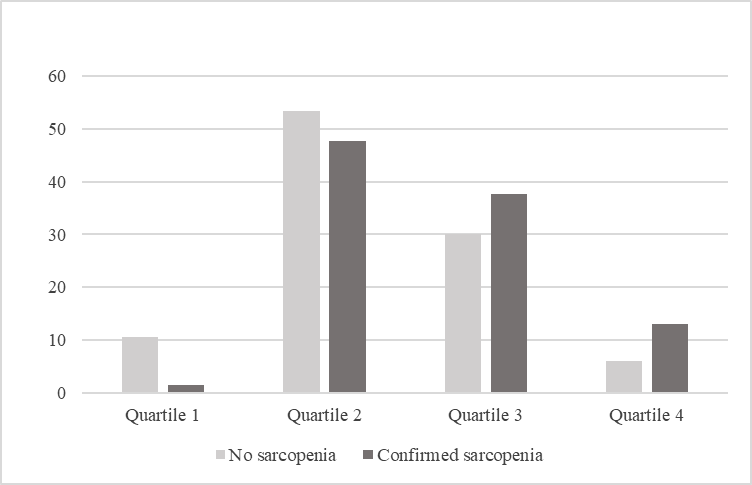


*

*

%

*FIM score Quartile 1 (n = 26):* 99 - 126 points; *FIM score Quartile 2 (n = 159):* 72 - 98 points; *FIM score* *Quartile 3 (n = 97):* 45 - 71 points; *FIM score* *Quartile 4 (n = 23):* 18 - 44 points.

* Significant difference (*p* <0.05) between the groups “no sarcopenia” and “confirmed sarcopenia”.

Abbreviations: FIM, functional independence measure
